# Interleukin-18 cytokine in immunity, inflammation, and autoimmunity: Biological role in induction, regulation, and treatment

**DOI:** 10.3389/fimmu.2022.919973

**Published:** 2022-08-11

**Authors:** Stella Amarachi Ihim, Sharafudeen Dahiru Abubakar, Zeineb Zian, Takanori Sasaki, Mohammad Saffarioun, Shayan Maleknia, Gholamreza Azizi

**Affiliations:** ^1^ Department of Molecular and Cellular Pharmacology, University of Shizuoka, Shizuoka, Japan; ^2^ Department of Pharmacology and Toxicology, University of Nigeria, Nsukka, Nigeria; ^3^ Department of Science Laboratory Technology, University of Nigeria, Nsukka, Nigeria; ^4^ Division of Molecular Pathology, Research Institute for Biomedical Sciences, Tokyo University of Science, Tokyo, Japan; ^5^ Department of Medical Laboratory Science, College of Medical Science, Ahmadu Bello University, Zaria, Nigeria; ^6^ Biomedical Genomics and Oncogenetics Research Laboratory, Faculty of Sciences and Techniques of Tangier, Abdelmalek Essaadi University, Tetouan, Morocco; ^7^ Division of Rheumatology, Immunology and Allergy, Department of Medicine, Brigham and Women’s Hospital, Harvard Medical School, Boston, MA, United States; ^8^ Division of Rheumatology, Department of Internal Medicine, Keio University School of Medicine, Tokyo, Japan; ^9^ Biopharmaceutical Research Center, AryoGen Pharmed Inc., Alborz University of Medical Sciences, Karaj, Iran; ^10^ Non-Communicable Diseases Research Center, Alborz University of Medical Sciences, Karaj, Iran

**Keywords:** inflammatory diseases, autoimmune diseases, interleukin-18, monoclonal antibodies, immunity

## Abstract

Interleukin-18 (IL-18) is a potent pro-inflammatory cytokine involved in host defense against infections and regulates the innate and acquired immune response. IL-18 is produced by both hematopoietic and non-hematopoietic cells, including monocytes, macrophages, keratinocytes and mesenchymal cell. IL-18 could potentially induce inflammatory and cytotoxic immune cell activities leading to autoimmunity. Its elevated levels have been reported in the blood of patients with some immune-related diseases, including rheumatoid arthritis, systemic lupus erythematosus, type I diabetes mellitus, atopic dermatitis, psoriasis, and inflammatory bowel disease. In the present review, we aimed to summarize the biological properties of IL-18 and its pathological role in different autoimmune diseases. We also reported some monoclonal antibodies and drugs targeting IL-18. Most of these monoclonal antibodies and drugs have only produced partial effectiveness or complete ineffectiveness *in vitro*, *in vivo* and human studies. The ineffectiveness of these drugs targeting IL-18 may be largely due to the loophole caused by the involvement of other cytokines and proteins in the signaling pathway of many inflammatory diseases besides the involvement of IL-18. Combination drug therapies, that focus on IL-18 inhibition, in addition to other cytokines, are highly recommended to be considered as an important area of research that needs to be explored.

## Introduction

Interleukin 18 belongs to the IL-1 family of cytokines, which is a group comprising 11 member cytokines that promote the activity of the innate immune system (1, 2). IL-18 stimulates both the innate immune and acquired immune responses. It acts on T helper 1 (Th1) cells, macrophages, Natural killer (NK) cells, natural killer T (NKT) cells, B cells, dendritic cells (DCs), and even non-polarized T cells to produce interferon gamma (IFN-γ) in the presence of IL-12. In the absence of IL-12, IL-18 with IL-2, induces type 2 T helper (Th2) cytokines from NK cells, NKT cells with a CD4^+^ phenotype, and even committed Th1 cells. Additionally, IL-18, in synergy with IL-3, induces basophils and mast cells to produce IL-4 and IL-13 (3, 4). IL-18 displays its pleiotropic action depending on its cytokine milieu suggesting its important pathophysiological role in health and disease (5).

The activity of IL-18 in both the innate and adaptive immune response implicates it in several inflammatory and autoimmune conditions (6). IL-18 levels are usually elevated in psoriasis (7), systemic lupus erythematosus (SLE) (8, 9), hypertension, chronic kidney disease (10), multiple sclerosis (MS) patients (11, 12) and Coronavirus disease 2019 (COVID-19) (13, 14) which correlates with caspase-1 levels (15). In a mouse model of autoimmune diabetes, IL-18 messenger RNA (mRNA) expression strongly correlated with destructive insulitis most likely due to IFN-γ secretion (16, 17). It was also shown that IL-18^-/-^ NOD mice developed less reactive islet cells than NOD wild type mice (18). To further strengthen its role in autoimmune diseases, IL18^-/-^ mice showed better disease outcome in Collagen-induced arthritis model in mice (19). In addition, IL18^-/-^ mice did not develop disease in animal models of experimental autoimmune encephalitis (20, 21) and experimental autoimmune myasthenia gravis (EAMG) (22). Gene expression analysis in pregnant women revealed more than four times higher expression of IL-18 in patients experiencing recurrent miscarriage (23). This clearly shows that IL-18 cytokine has a role in the progression or development of some inflammatory and autoimmune diseases.

Since the introduction of the hybridoma technique in 1975 by Kohler and Milstein (24), more than 550 therapeutic antibodies targeting specific antigens have been studied and at least 70 approved for clinical use (25). Generation of humanized antibodies (26) brought about great advances in this field (27). These antibodies and other biologics can bind to their antigens with high fidelity and affect disease processes or by modulating immune responses (28) and hence, provide powerful tools in the management of chronic conditions such as cancer and autoimmune diseases (29).

In this review, we will provide some information about the production, activation, signaling, and pathophysiology of IL-18. We will also report and discuss the used monoclonal antibodies, inhibitors, and drugs targeting this cytokine.

## Interleukin 18 characteristics

### IL-18 and other related cytokines

IL-18 was initially called “IFN-γ‐inducing factor” (30) due to its action to induce IFN-γ secretion in CD3-stimulated Th1 cells leading to liver toxicity. Subsequently, it was refined from mouse liver cells treated with *Proprionibacterium acnes* and lipopolysaccharides (LPS), with the isolate then renamed IL-18 (31, 32). At first, the liver toxicity resulting from the induction of IFN-γ secretion was attributed to IL-12, but was found to be prevented by an IL-18 antibody. Further research showed that IL-18 deficient mice primed with LPS did not develop liver damage (33, 34). The mechanism for the liver toxicity was found out to be the induction of Fas ligand (FasL) expression and tumor necrosis factor alpha (TNF-α) production in liver NK cells (33–36). Human IL-18 is a 193 amino acid-protein while mouse IL-18 consist of 192 amino acids (30, 37).

Unexpectedly, IL-18 was found to share similarities with IL-1β and the IL-1 family in 4 ways: 1) homology in the amino acid sequence 2) they share a common β-pleated sheet structure, 3) they are all secreted as an inactive precursor, and 4) they have similar signaling pathways (3, 38–40). Apart from these similarities, other features between IL-18 and IL-1β are remarkably distinctive. As the name indicates, IL-1 was the first member of the family to be discovered and it has been extensively studied in various immune processes and disease conditions (1).

The receptors of the IL-1 family contain an extracellular immunoglobulin domain and a Toll/IL-1 receptor (TIR) cytoplasmic domain. Binding of the ligand to the appropriate receptor recruits a second receptor subunit. The receptor heterodimer formed and the alignment of two TIR domains facilitates the recruitment of myeloid differentiation primary response protein 88 (Myd88), IL-1R-associated kinase 4 (IRAK4), TNFR-associated factor 6 (TRAF6), and other signaling molecules. The interaction usually engages the activation of the nuclear factor-κB (NF-κB) and mitogen-activated protein kinase (MAPK) pathways (41–43).

The members of the IL-1 family of cytokines are released in response to Toll like receptor (TLR) signaling to amplify the danger messages to other cells that cannot recognize microbial antigens (due to the lack of certain receptors). Basically, these cytokines stimulate the innate immune system and serve a critical link between innate immune responses and the appropriate adaptive immune response. The members of the IL-1 family, IL-18 inclusive, are produced by neutrophils, monocytes, and macrophages. The IL-1 family members also respond to stimulation by IL-1, IL-18, and IL-33 (44–47).

### Cell source, production and activation of IL-18

Unlike members of the IL-1 family of cytokines, IL‐18 gene in humans is located on chromosome 11, and in mice it is found on chromosome 9. The gene contains 7 exons with two distinct promoters on exon 1 and 2 including an interferon consensus sequence binding protein and a PU.1 binding sites (a hematopoietic-specific transcription factor) (3). Another defining feature of the IL-18 gene unlike other cytokine genes, is that it has few RNA‐destabilizing elements, and this translates to an unusually stable cytokine expression. IL‐18 gene encodes for a 193 amino acid-24 kDa inactive precursor localized in the cell cytoplasm. TLR binding of Pathogen associated molecular patterns (PAMPs) and activation of the NF‐kB pathway induces transcription of IL‐18 precursor (48).

**Figure 1:**
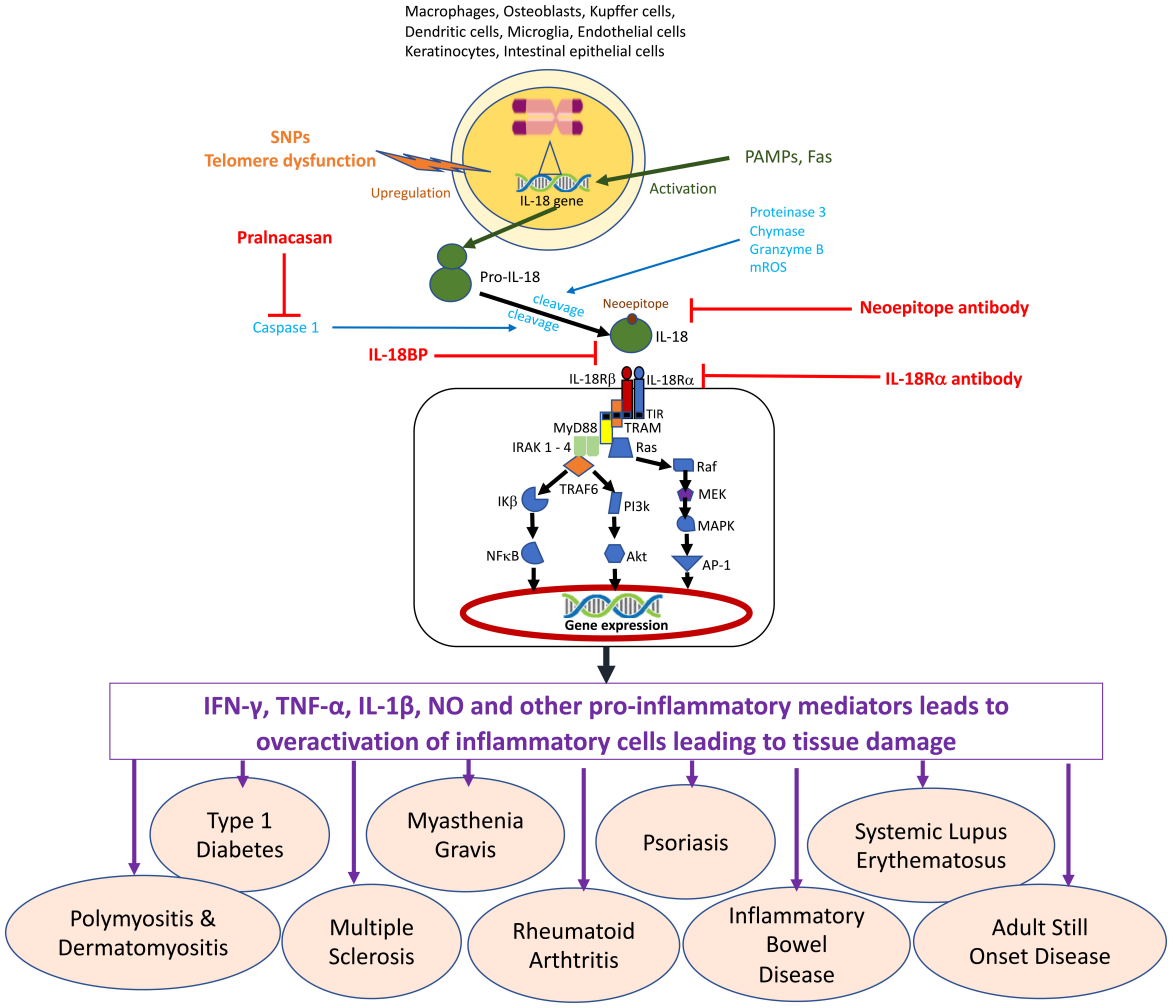
The cell sources and signaling mechanism for Interleukin 18.

Many cell types are capable of producing IL-18 including hematopoietic and non-hematopoietic cells (**Figure 1**). It was originally thought to be secreted only by Kupffer cells and liver-resident macrophages at resting stage. The IL‐18 precursor is constitutively produced in circulating monocytes, resident macrophages, and DCs, unlike IL‐1β which is not found in healthy individuals (39, 49). The IL‐18 precursor is also found to be released by most endothelial cells, keratinocytes, osteoblasts, most intestinal epithelial cells, and mesenchymal cells (48). IL‐18 can also be discharged in its precursor form from dead cells which can be acted upon by neutrophil proteases such as proteinase 3 (50) into its active form.

As with other members of the IL-1 family, IL-18 cytokine is produced in the cytoplasm as an inactive precursor named pro-IL-18. To be secreted, it requires proteolytic processing into biologically active IL-18 (3, 30, 51). It was further revealed that cleavage of pro-IL-18 (just like pro-IL-1β) into mature IL-18, relied on the action of caspase-1, an intracellular cysteine protease produced in the NACHT-LRR and pyrin domain-containing protein 3 (NLRP3) inflammasome. This complex consists of NLRP3, pro-caspase-1, and apoptosis-associated speck-like protein containing a caspase recruitment domain (adaptor molecule ASC) (52–55). Caspase 1 can be activated by several established inflammasomes and could belong either to the AIM2‐like receptors, Nod‐like receptors, or the TRIM family containing either a PYD or a CARD domain (56). The activation of Caspase 1 results in pyroptosis - a cell‐death program, which prompts the membrane pores formation and the release of mature IL‐1β and IL‐18 (5, 48). This is why many researchers use IL-18 as a marker of inflammasome activation (57)

It is widely believed that other caspase-mediated pathways are involved in IL-18 production because treatment with FasL led to stimulation of Fas-expressing macrophages to produce active IL-18 independent of caspase-1 involvement (58). It was showed that caspase-8 was involved in the Fas mediated noncanonical IL-1β and IL-18 maturation (59). Proteases that can activate IL-18 without the contribution of the inflammasome include proteinase 3 (50), chymase (60), and granzyme B (61). Recently, mitochondrial reactive oxygen species (mROS) have been shown to be critical in T-cell receptor (TCR) independent activation of IL-18 *via* downstream activation of STAT4 and NFkB which is regulated by Fas/FasL signaling (62)

### IL-18 signaling

IL-18 receptor (IL-18R) is requisite for IL-18 signaling. IL-18R is expressed in T cells and NK cells which is essential for IFN-γ production *via* STAT4 (Signal transducer and activator of transcription 4) signaling (63–66). It is also expressed by non-immune cells such as neurons and epithelial cells but in this case, it is involved in cell differentiation and survival (5).

The IL-18R is composed of 2 subunits - IL-18Rα chain (which is inducible and also called IL-1R-related protein or IL-1R5) and IL-18Rβ chain (which is constitutively expressed and also called IL-1R-associated protein-like or IL-R7) (3, 48). The IL-18Rα and IL-18Rβ chains are constitutive members of the IL-1R family and their cytoplasmic domains contain a TIR domain, which is shared by the TLRs (1, 3, 45, 48, 67). When triggered by IL-18, IL-18Rα forms a high-affinity binding heterodimer with IL-18Rβ that facilitates downstream signal transduction (68, 69). The TIR domains of the formed IL-18R complex (IL-18/IL-18Rα/IL-18Rβ) recruits MyD88, a signal adaptor which contains a TIR domain, thereby creating a TIR–TIR interface (1, 3, 45, 48, 67, 70) which is critical for IL-18 signaling (70). MyD88 binding subsequently recruits IRAK1, IRAK4, and TRAF6 (70). Upon binding to TRAF6, inhibitor of κB (IκB) is deactivated, this allows phosphorylated p65/p50 to translocate into the nucleus (71), which results in the activation of NFkB. Also activated are Extracellular signal-regulated Kinase (ERK), and c-jun N-terminal kinase (JNK), which make up the MAPK cascade. Eventually, these lead to the expression of appropriate genes such as Il-4, Il-13, IFN-γ, and TNF, which facilitates cell differentiation/survival, and FasL which facilitates apoptosis (33, 35, 72).

IL-18 stimulation phosphorylates and activates the members of the phosphatidylinositol-3 kinase (PI3K) family such as mammalian target of rapamycin (mTOR) and the expressions of Bcl-xL and Bcl2 (73, 74). PI3K and its pathway in myeloid cells is known to suppress inflammatory cytokine production *via* TLR signaling (75), but in this case, IL-18 stimulation increases the proliferation and survival of both immune and non-immune cells such as NK cells, keratinocytes, and neurons (76, 77). In neutrophils, the p38 MAP kinase pathway has been shown to be stimulated by the IL‐18/IL‐18Rα/IL‐18Rβ complex. This complex was also demonstrated to phosphorylate STAT3 in NK and hippocampal cell lines (78–80).

IL-18-dependent cell activation can be inhibited by the naturally occurring IL-18-binding protein (IL-18BP) (81) due to its high affinity for IL-18. Its binding leads to a downregulation of IL-18-induced cell responses, such as IFN-γ production. IL-37, an anti-inflammatory cytokine and a member of the IL-1 family of cytokines is another inhibitor of IL-18 (82), which binds to IL-18Rα with low affinity. This interaction recruits IL-1R8 (SIGIRR), an orphan receptor to form an IL-37/IL-18Rα/IL-1R8 complex. This complex cannot bind to MyD88 and hence cannot recruit IL-18Rβ, thereby obliterating signal transduction *via* IL-18R, but instead induces an anti-inflammatory signal into the cell (4, 48).

### Physiological role of IL-18 in immune cells and host defense

In order to trigger the innate immune system, IL-18 combines with IL-12, stimulating NK cells to respond to cancers and infections. Hence, IL-18 and IL-12 play a key role in enhancing NK cell activities. The vital role played by IL-18 in establishing NK cell activity has been demonstrated in IL-18 deficient mice, which have increased susceptibility to infection and impaired NK cell activity (83). The combined stimulation with IL-18, IL-15, and IL-12 has also been linked with the generation of memory-like NK cells (84). Similarly, IL-18 together with IL-12 can activate macrophages, which can produce IFN-γ (85). Other cell types which are capable of producing IFN-γ *via* the synergistic action of IL-18 and IL-12 include non-polarized T cells, Th1 cells, DCs, and B cells (4)

In the adaptive immune system, IL-18 promotes activation and the differentiation of T cells (48). IL-18 is able to upregulate the production of IFN-γ, which is required for host defense. To induce marked IFN-γ production, IL-18 requires IL-12 or IL-15 as a surrounding cytokine in synergy since they increase the expression of IL-18Rα. *In vitro*, the production of IFN-γ by IL-18 in combination with IL-12 occurs by acting on NF-kB in CD4+ and CD8+ T cells (3). Nevertheless, in the absence of IL-12, IL-18 induces the secretion of IL-2 and IL-13 as well as a little amount of IFN-γ in T cells stimulated with anti-CD3 antibody (86). In Th1 cells, IL-18 promotes the expression of IL-12Rβ2 while IL-12 upregulates IL-18R expression (87). IL-18 acts as a co-stimulant to amplify the production of IFN-γ, Granulocyte-Macrophage Colony Stimulating Factor (GM-CSF), IL-2, and IL-2Rα by Th1 cells but not Th2 cells. Hence, IL-18 cannot act on Th2 cells (86).

IL-18 also upregulates the cytotoxic activities of NK and CD8 T cells by killing target cells through cytotoxic molecules such as perforin or by inducing apoptosis using Fas-expressing target cells (88–90). It induces IL-4 production and may be implicated in the induction of allergic inflammation (91). *In vivo*, IL-18 remains a potent IFN-γ inducing factor (68) and it is highly essential for the development of NK cells but does not play a major role in the development of Th1 cells (33). It has been observed that the IL-18 mediated Th1 cell amplifying actions contributes to microbial resistance (92).

Independent of IL-12 or IL-15, IL-18 also plays a vital role as a principal protective agent in host defense. The complement system and antibodies principally respond to extracellular microbes while T and NK cells eliminate intracellular microbes. Therefore, protection against intracellular microbes is majorly dependent on NK and T cells especially Th1 that produces IFN-γ. IFN-γ is critically important in eliminating microbes *via* the activation of type 2 nitric oxide synthase (NOS2) (86). In a mouse model of disseminated intracellular bacterium - *Mycobacterium avium* infection, IL-18 has been shown to be valuable in offering protective immunity to mycobacteria through IFN-γ induction (93). Another study also observed similar expression of IL-18 and IFN-γ in children with severe Mycoplasma pneumoniae pneumonia (94). In another study in mice infected with *Leishmania major* (an intracellular protozoon), daily administration of IL-12 and IL-18 offered protective immunity against reinfection and inhibited the expansion of *L. major* infection (92).

In addition, IL-18 may play a potent role in activating CD8+ T cells, which have activity against viral infection. In mouse model infected with Herpes simplex virus (HSV), IL-18 has a protective role against viral infection (95). Epstein-Barr virus (EBV) was also showed to activate mucosal-associated invariant T cell, which were found to be a potent source of IL-18. Its dysregulation has been theorized as a mechanism for EBV associated T/NKT cell lymphoproliferative disease (96). IL-18 in collaboration with IL-12 may be involved in inhibiting IgE production (97) in an IFN-γ-dependent manner but IL-18 alone induces *in vivo* IgE accumulation (91). In SJL mice, IL-18 suppressed IgE production following helminth infection. The weakened IgE production was restored by the administration of anti-IL-12 and IL-18, signifying active suppression by macrophages that secrete IL-12 and IL-18 (98). Finally, IL-18, independent of IFN-γ or other cytokines, shows the characteristics of other pro-inflammatory cytokines. Some of these activities include increased cell adhesion molecule (CAM), production of chemokines and synthesis of NO (91). IL-18 has also been shown to contribute to host defense and inflammation in the Paneth cells of the intestinal mucosa in an IL-22 dependent manner *via* STAT3 signaling (99).

IL-18 might also play a role in tumor control and cancer chemotherapy/immunotherapy. It has been shown that cytotoxic drugs like doxorubicin, paclitaxel, topotecan, carboplatin and gemcitabine selectively co-operate with IL-18 to improve anti-tumor effectiveness (100, 101). Moreover, tumor-derived IL-18BP is an immune checkpoint molecule that inhibits IL-18 mediated anti-tumor activity in mouse and human tumors. An engineered decoy-resistant IL-18 restored IL-18 signaling and subsequent anti-tumor activity (102, 103). Not only does the cytokine improve anti-tumor activity; IL-18 and IL-12 transduced DCs also showed better recruitment of CD4 and CD8 T cells into tumor microenvironment in mouse colorectal tumor models leading to inhibition of tumor growth (104, 105)

## Pathophysiological autoimmune conditions associated with IL-18

The broad biological role of the IL-18 cytokine on immune cells have revealed its potential role in inflammatory and autoimmune diseases. IL-18 have been shown to be a diagnostic marker and predictor of inflammatory conditions like myocardial ischemia (106), acute respiratory distress syndrome (107, 108), chronic obstructive pulmonary disease (109), post infectious bronchiolitis obliterans (110) and sepsis-induced multi organ injury (111). IL-18 has also received increase attention in the pathophysiology of neuro-vascular diseases such as in intracerebral hemorrhage (112), Japanese encephalitis (113), ocular Behcet disease (114), abdominal aortic aneurysm (115), amyotrophic lateral sclerosis (116), glioma (117) and most interestingly in cognitive impairment (118) and agitation in severe mental disease (119).

Here, we mentioned the contribution of IL-18 to several autoimmune diseases. **Table 1** provides a brief overview of the mechanisms of involvement of IL-18 in some autoimmune diseases and **Table 2** shows some clinical studies indicating the pathogenic role of IL-18 in inflammatory diseases.

**Table 1:** Immunopathogenic mechanism of interleukin 18 involvement in autoimmune diseases.

No	Disease examined	Alterations of IL-18	Immunopathogenic mechanism of interleukin 18 in disease	References
1	Type 1 diabetes	Serum IL-18 levels are increased in T1D patients compared to control.	Inflammatory cells may invade islets, destroy β cells and release cytokines TNF, IL-1β, and IFN-γ leading to apoptosis. IFN-γ may also be induced by IL-18, further promoting β cell apoptosis.	(120)
2	Multiple sclerosis	IL-18 serum levels in MS patients are considerably higher than that in healthy individuals	IL-18 may play a role in the disease by driving these inflammatory responses, hence neuronal damage. The involvement of IL-18 in MS is acknowledged but the exact mechanisms remain unknown.	(121, 122)
3	Myasthenia gravis	IL-18 levels are higher in generalized MG than other control individuals including healthy subjects.	It is suggested that IL-18 affects MG through its role in IFN-γ secretion and IL-12-dependent Th1 phenotype polarization, which are strongly involved in the generation of immunopathogenic auto-antibodies at the neuro-muscular junction in MG.	(123)
4	Inflammatory bowel disease	IL-18 levels are elevated in patients with IBD compared to healthy individuals.	IL-1 and Il-18 may mediate inflammatory cascade by inducing increased IL-18 RNA and protein as emphasized by clinical samples although relatively little is known.	(124)
5	Rheumatoid arthritis	Serum IL-18 levels are increased in RA patients compared to normal healthy subjects.	IL-18 may play a vital role in RA by inducing synovial fibroblast to upregulate expression of CXC chemokines *via* NFκB. This ultimately places IL-18 in a strategic role for promoting synovial inflammation	(125)
6	Psoriasis	Compared with controls, patients with Psoriasis have higher serum concentration of IL-18	It is suggested that keratinocytes derived IL-18 might be involved in the dermal Th1-type immune response involved in psoriatic lesions	(126, 127)
7	Systemic lupus erythematosus	Patients with SLE show significantly higher levels of circulating IL-18 compared to healthy controls	The mechanism is still unknown, but it is thought the IL-18 promotes SLE pathogenesis by its critical role in the inflammatory response	(128)
8	Adult-Onset Still’s Disease	Elevated serum IL-18 levels compared with controls and other disease conditions	Pathophysiology of disease is still unclear but IL-18 and other IL-1 family secreting inflammatory cells are implicated in the pathogenesis	(129)
9	Poliomyositis and Dermatomyositis	Serum IL-18 levels correlated with disease activity and progression	Autoantibodies stimulate complement and dendritic cells which in turn activate IL-18 secreting autoreactive T and B cells	(130)

**Table 2:** Clinical studies showing the pathogenic role of IL-18 in inflammatory diseases. .

No	Disease examined	Study design	Result/conclusion	References
1	Type 1 diabetes	Serum levels of IL-18 and other mediators was estimated in 35 type 1 diabetic patients and their relatives who share HLA diabetic susceptibility genes, and 31 healthy volunteers	IL-18 and other mediator levels was elevated in subjects with type 1 diabetes and their first-degree relatives, who share diabetic HLA haplotypes	(131)
2	Type 1 diabetes	IL-18 levels in the plasma of 26 juveniles with type 1 diabetes (T1D) in comparison to 36 control healthy volunteers was analyzed	IL-18 levels were significantly elevated in patients with T1D, compared to control subjects. Two negative regulators of IL-18 function, IL-18 binding protein (IL-18BP) and IL-37 remained unchanged	(132)
3	Type 1 diabetes	In the sera from 65 diabetic [30 with type 1 insulin dependent diabetes mellitus (IDDM) and 35 with type 2 non-insulin dependent diabetes mellitus (NIDDM)] patients and 15 healthy volunteers, Il-18 levels were measured	In both IDDM and NIDDM individuals as compared to the control group, IL-18 levels were higher	(133)
4	Multiple sclerosis	110 MS patients and 110 healthy individuals were recruited and Il-18 serum levels and polymorphism were measured	There was a significantly higher IL-18 serum level and different frequencies of two polymorphisms of IL-18 in MS patients	(122)
5	Multiple sclerosis	To determine whether there was any relationship between IL18 gene polymorphisms and MS, IL18 genotyping were performed in 101 MS patients and 164 control subjects	IL18 gene polymorphisms at position -137 might be a genetic risk factor for MS in the Turkish population.	(134)
6	Multiple sclerosis	IL-18 levels were determined in 30 non treated Relapsing–remitting (RR)-MS patients and compared to 30 IFN-β-treated MS patients	Naïve MS patients showed significantly higher levels of interleukin-18	(121)
7	Myasthenia gravis	IL-18 levels were determined in generalized MG patients compared to ocular myasthenia gravis patients	IL-18 levels were higher in generalized than in ocular myasthenia	(123)
8	Inflammatory bowel disease	The correlation of IL-18 and IL-18BP with disease activity and other disease parameters in inflammatory bowel disease was investigated by measuring IL-18 and IL-18BP isoform in 129 patients and 10 healthy individuals	IL-18 and IL-18BP levels are higher in patients with inflammatory bowel disease compared to healthy individuals	(124)
9	Inflammatory bowel disease	changes in serum IL-18 concentrations in patients with IBD was compared with 21 control subjects	Serum IL-18 concentrations in 5 IBD patients were 1.7 times higher than concentrations in control subjects	(135)
10	Inflammatory bowel disease	Serum IL-18 measurements was obtained in 41 children with IBD and 32 non-IBD control groups.	Serum IL-18, measured by ELISA, was elevated in children with IBD compared to the control group	(136)
11	Rheumatoid arthritis	serum pro-inflammatory profiles of IL-18 in 78 female rheumatoid arthritis (RA) patients was compared with 51 healthy women to establish the relative importance of pro-inflammatory cytokines	The cytokine IL-18 assayed was 2.3 folds significantly elevated in the sera of RA female patients than healthy controls.	(137)
12	Rheumatoid arthritis	The serum levels of IL-18 in 140 RA patients were compared with40 healthy control to ascertain the severity and treatment of RA patients if there are any correlations	IL-18 level was significantly elevated in the sera of RA patients than healthy controls	(125)
13	Rheumatoid arthritis	The expression patterns of IL-18 in synovial biopsy tissue of 29 patients with active RA was determined	IL-18 was detectable in 80% of the RA patients, in both the lining and sublining of the knee synovial tissue	(138)
14	Psoriasis	Biopsies were taken from a psoriatic lesion (large plaque type) of four psoriasis patients and from the skin of four normal healthy individuals	The expression of IL-18 was increased in psoriatic lesional skin relative to that in normal skin.	(126)
15	Psoriasis	Gingival crevicular fluid (GCF) levels of IL-18 in 42 psoriatic patients and 39 healthy controls were compared	Psoriasis was associated with elevated IL-18 compared to healthy controls	(139)
16	Psoriasis	serum samples from 36 patients with psoriasis and 156 healthy controls were compared	IL-18 are elevated in the serum of patients with psoriasis compared with control	(140)
17	Systemic lupus disease	Serum IL-18 levels were compared in 184 SLE patient and 52 control subjects	Serum IL-18 levels were statistically significantly higher in SLE patients compared to healthy controls	(141)
18	Systemic lupus disease	Clinical evaluation of total and free IL-18 was carried out in 74 active SLE patients and compared with SLE inactive control	Total and free IL-18 were higher in patients with active vs. inactive disease.	(142)
19	Systemic lupus disease	serum IL-18 were collected at time of disease onset and 6 months after treatment in paediatric SLE patients (pSLE)	The role of serum IL-18 as biomarker and status of renal flares among pSLE population was shown.	(143)
20	Adult-onset still’s disease	Free IL-18 Serum levels of 37 AOSD patients and 138 controls were compared	Free IL-18 was significantly higher in AOSD patients compared to control	(129)
21	Adult-onset still’s disease	Serum levels of IL-18 were measured in 21 patients with AOSD	Circulating IL-18 levels were significantly higher in those with active disease compared with 85 controls	(144)
22	Polymyositis and Dermatomyositis	IL-18 levels were determined in two cohorts of patients. In cohort one,10 new-onset myositis patients (IL-18 expression was compared between symptomatic and asymptomatic muscle biopsies that were taken prior to treatment). The second cohort consisted of another 10 patients with repeated muscle biopsies before and after 8 months with conventional immunosuppressive treatment.	Total IL-18 expression in muscle tissues from the new-onset patients, at both symptomatic and asymptomatic sites, was significantly higher compared with healthy controls	(145)

### Type 1 diabetes (T1D)

The autoimmune destruction of the host pancreatic β cells that produce insulin results in a chronic disease named Type 1 diabetes (T1D). It was suggested that inflammatory cells may invade islets, destroy β cells, and release cytokines including TNF-α, IL-1β, and IFN-γ leading to pancreatic β cells apoptosis (132). It has recently been shown that IL-18 actually maintains islet β cells function and homeostasis. IL-18 expressed on islet α cells, IL-18R on acinar cells and Na-Cl co-transporter (NCC) on β cells play a role in this homeostasis. A deficiency in NCC on β cells or IL-18R in acinar cells reduces β cell proliferation and islet size with a concomitant rise in β cell apoptosis and exocrine macrophage accumulation (146). T1D is possibly a Th1 cell-mediated disease (147) that affects millions of people around the world and its etiology is complex with a combination of genetic and environmental pathogenic factors (148, 149). Although the Human leukocyte antigen (HLA) genes are known to have a role in the development of T1D, evidence have continued to indicate that the pro-inflammatory IL-18 contributes to the genetic susceptibility to T1D (150). Studies in both humans and mice have revealed that IL-18 genes are localized in chromosome areas associated with T1D susceptibility (151). Comprising of six exons and five introns, the IL-18 gene is located on chromosome 11q22.2-q22.3 (152). Several IL-18 polymorphisms have been identified, however the genetic relationship between single nucleotide polymorphisms (SNPs) at positions-137, -607 in IL-18 gene promoter and T1D have been of interest and is widely reported in previous studies (153). Conversely, other studies failed to show any association between T1D and these SNPs. A subclinical early report showed that serum IL-18 levels were elevated in first degree relatives of T1D patients, indicating a predictive role of IL-18 in human diseases (131). Many other studies have indicated that increased serum IL-18 levels in patients was associated with elevated glycated hemoglobin (HbA1C), which might indicate a relation between hyperglycemia and IL-18 (132). Higher serum IL-18 levels have also been linked with diabetic ketoacidosis (6, 120) and nephropathy (133). It was found that elevated IL-18 mRNA production by macrophages and a subsequent increase in IFN-γ circulating levels was correlated with an active stage of auto-immune diabetes in non-obese diabetic (NOD) mice (a well-established model for the study of autoimmune diabetes) (154). Furthermore, progression from benign to destructive insulitis have been linked with IL-18 mRNA in NOD mice (16). The administration of IL-18 *via* IL-18 expressing plasmid delivery to 4 weeks old NOD mice promoted the development of insulitis/diabetes (154). However, when IL-18 was administered to 10 weeks old female NOD mice, exogenously, the mice were protected from diabetes (155). Since IL-18 regulates Th1 and Th2 responses based on the cytokine present in its environment, the contrast aforementioned may be attributed to this dual role of IL-18 (86). Moreover, in another animal model of diabetes, short-term prophylactic treatment with inhibitors of IL-18 (IL-18Bp-Fc-fusion molecule) significantly protected animals from developing overt diabetes, further strengthening the evidence suggesting the role of IL-18 in T1D (156). Similar findings were observed after treatment with HIV-1 protease inhibitors in rats (157). IL-18 has also been shown to be a mediator of polycystic ovarian syndrome – a condition strongly linked to T1D (158, 159). Taken together, the results of these numerous animal and human studies suggest that IL-18 may play a pathogenic role in T1D through its interferon gamma (IFN- γ)-inducing potential. This, therefore, opens the possibility of IL-18 being a potential therapeutic target.

### Multiple sclerosis (MS)

This is a chronic progressive autoimmune disease wherein the immune system attacks and destroys the protective myelin sheaths over the nerve fibers in the central nervous system (CNS). Inflammation plays a vital role in the advancement of MS. It is characterized by neuro-inflammatory and neuro-degenerative processes leading to the activation of auto-immune T cells and “CNS macrophages” - microglia that enhance a pathological immune response called cytokine storm (121). The SNPs- rs1946518, rs360719, and rs187238 have also been implicated in patients with MS patients (122) with high serum IL-18 levels. IL-18 and IL-1β may drive these inflammatory responses. Inflammasome is a complex intracellular receptors and stressor sensors that activate inflammatory signaling pathways vital for host defense (160). However, the dysregulation of the inflammasome can result to auto-inflammatory and auto-immune disorders (121). The NLRP3 inflammasome has been established as a critical contributor of neuro-inflammation and drives the activation of caspase 1 and the processing of IL-1β and IL-18, which subsequently mediates immune cascade responses (161). As in human MS, experimental autoimmune encephalomyelitis (EAE) in mice is also characterized by demyelinating inflammation induced by immunization with antigens (such as myelin basic protein-MBP) which serves an equivalent analogy (162). Blood caspase1 mRNA levels were increased in EAE mice which correlated with the severity of MS. This result corroborated well with the studies in humans, wherein amplified Caspase-1 levels were observed in the cerebrospinal fluid of patients with acute MS (15). In IL-18-/- mice, antibodies blocking IL-18Rα caused mice to be MS-resistant implying the existence of IL-18Rα ligands and IL-18^-/-^ (6). It was reported that spleen cells from IL-18Rα-/- mice yielded considerably greater amount of pro-inflammatory cytokines in comparison to those from wild type or IL-18^-/-^ mice in response to concanavalin A (con A) stimulation (163). Serum levels of IL-18 were the highest levels in patients with chronic MS compared to those relapsing-remitting MS – both acute and stable (11). Similarly, mRNA and protein levels of caspase 1 and IL-18 were identified in peripheral blood mononuclear cells in MS patients who were never administered immunomodulatory drugs (164). In summary, while data from EAE model suggests a role for IL-18R, data from acute relapsing MS patients suggests a role for IL-18 in disease advancement. This may mean that aside from IL-18, other ligands may be involved.

### Myasthenia gravis (MG)

Myasthenia gravis (MG) is an autoantibody-mediated disease affecting the neuro-muscular junctions (NMJs). It is mediated by auto-antibodies against nicotinic AchR (acetylcholine receptor) (123). The EAMG model in mice or rats is a disease model mimicking the clinical and immunopathological characteristics of human MG (165, 166). Disease progression in both humans and mice is dependent on the production of reactive autoantibodies at the NMJ by B cells. Activated T cells help B cells proliferate and differentiate by secreting IL-12 and IFN-γ. Since IFN-γ is involved in the development of EAMG in both acute and chronic MG stages, blocking IL-18 with anti-IL-18 antibodies or disrupting CD40-CD40L interaction was found to suppress MG severity (165). The mechanism appears to be by the regulation of Th1 and CD40L levels and upregulation of TGF-β and CTLA-4 (167). Moreover, in MG patients treated with immunosuppressive drugs, serum IL-18 levels were considerably diminished (123, 168). Overall, there are only a few clinical studies showing the role played by IL-18 in contributing to the pathology of MG. This is an area that still needs to be explored.

### Inflammatory bowel disease (IBD)

Studies have pointed the role played by IL-18 in Inflammatory bowel disease (IBD) by promoting intestinal homeostatic auto-inflammatory responses or protecting against the breach of pathogens through the epithelial barrier (169). The SNP– IL-18 rs1946518 has been shown to be a predisposing factor to IBD development (170). IBD is a chronic complex autoimmune disease characterized by the inflammation of the intestinal mucosa. This disease may be subclassified into Crohn’s disease (CD) and ulcerative colitis which have dissimilar clinical manifestations but similarly characterized by chronic relapsing pathogenic inflammation and intestinal epithelial cell injury (171). The role of IL-18 in IBD may be primarily related to its place in regulating pro-inflammatory responses. Following inflammasome activation, pro-IL-18 promotes the production of IFN-γ, NK cell cytotoxicity and Th1 cell differentiation (172). The mitogen-activated protein kinase 2 (MAP3K2) is necessary for the IL-18-Th1 mediated intestinal inflammation (173) *via* the IL-18-MAP3K2-JNK axis. It was recently shown that telomere dysfunction drives ataxia-telegientaxia mutated (ATM) activation of the transcriptional factor YAP1 thereby upregulating pro-IL-18 which when stimulated by caspase 1 due to colonic microbiome drives IL-18 signaling (174). Pharmacological reactivation of telomerase activity was able to control the ATM/YAP1/pro-IL-18 axis (175). Interestingly, a deficiency in NLRP6 inflammasome, a known regulator of colonic homeostasis predominantly found in intestinal epithelial cells, is detrimental in dextran sodium sulfate (DSS) induced colitis (DSS is a frequently used mouse model for colitis in which drinking water spiked with DSS injures the intestinal epithelium like IL-18 deficiency) (169). In agreement, the favorable role of IL-18 inhibition using neutralizing anti-IL-18 antibodies or IL-18 Binding Protein have been reported in DSS or Trinitrobenzene sodium (TSNBS) induced models of IBD (176, 177). A study has shown that mice were prevented from developing DSS-induced colitis and mucosa damage when IL-18R was deleted (171). Double knock out of IL-1β and IL-18 cytokines increased transgenic mice protection from TNBS colitis induction compared to deletion of either cytokine (178). In clinical studies, elevated secretion of IL-18 has been linked with IBD severity (179). Serum IL-18 concentration was considerably increased in patients with Crohn’s disease than healthy patients suggesting that infiltrated macrophages in the inflamed intestinal mucosa produced IL-18 which then potentially regulate intestinal mucosa lymphocytes (136, 180). A Mendelian randomization study has positioned anit-IL-18 therapy to be useful for managing IBD (181). This assertion was further strengthened in a study that found that Crohn’s disease patients resistant to anti-TNF therapy had genetically susceptible IL-18 SNP and high serum IL-18 level (182).

### Rheumatoid arthritis (RA)

Several studies suggest that Rheumatoid arthritis (RA) is a Th1-driven systemic inflammatory disease of the synovial joints. SNP- IL-18 rs1946518 (−607 A > C), and IL-18 rs187238 (−137 G > C) in the IL-18 gene have been correlated with RA in certain populations (183, 184). In cell cultures using synovium from patients who had undergone synovectomy or total knee replacement surgery, results demonstrate the role of IL-18 and the expression of CXC chemokines by fibroblasts *via* NFκB signaling (185). In RA, IL-18 cytokine may contribute to inflammation by leukocyte extravasation *via* the upregulation of endothelial cell adhesion molecules, act directly as monocyte, neutrophil chemoattractant or lymphocyte and release chemokines from synovial fibroblasts (186, 187). The administration of IL-18 to mice with collagen induced arthritis (CIA) or incomplete Freund’s adjuvant immunized mice facilitated the development and severity of inflammation of cartilage (188, 189). This result was similar to that obtained when IL-18^-/-^ mice received IL-18 (19). In contrast, low levels of IFN-γ have been detected in RA synovitis (190). This is explained to occur because IL-18 sustains the Th1 phenotype but does not induce levels of IFN-γ production in the presence of elevated expression of inhibitory molecules such as IL-10 and TGF-β. The unique role of IL-18 in inducing the discharge and upregulation of angiogenic factors such as SDF-1α, MCP-1 and VEGF in RA synovial tissues *via* distinct pathways have been described (191). Similarly, the use of IL-18 to stimulate RA synovial fibroblasts *in vitro* induced the expression of surface vascular CAM and neutrophil chemoattractant (192); more IL-18 is then produced by synovial fibroblasts and through the action of TNF-α produced by synovial macrophages in a positive feedback mechanism. Significantly higher levels of IL-18 mRNA and protein was detected in RA synovial tissues but not osteoarthritis patients experiencing age related joint disorder (193). In systemic juvenile idiopathic arthritis, elevated IL-18 levels are also a hallmark of the disease. Impaired IL-18 signaling in NK cells were implicated with a dysregulated phosphorylation of the MAPK and NFkB pathways (194, 195).

### Psoriasis

Although its etiology is unknown, Psoriasis is known to be a chronic inflammatory skin condition suggested to involve a multifaceted plethora of cytokines and chemokines secreted by immune cells and other tissue cells (196, 197). The role of IL-18 in stimulating Th1 cells which produce IFN-γ-mediated inflammation in psoriatic lesions have been described (6). Human keratinocytes are able to produce IL-18. Hence, it is proposed that IL-18 secreted by keratinocytes might be involved in the dermal Th1 immune response involved in psoriatic lesion (126). In an IL-18 knockout mouse model of psoriasis induced by Imiquimod (IMQ), IMQ induced mice manifested larger areas of Munro micro abscesses and had upregulated expression of IL-1β, IL-4, and IL-27 compared to wild type (WT) (196). This indicates that IL-18 may exacerbate psoriatic inflammation and influence its pathology. Similarly, in human studies, skin sections of psoriasis patients showed elevated levels of IL-18 and Caspase-1 compared to healthy subjects (126). Furthermore, psoriasis patients serum revealed high levels of circulating IL-18 (7). The stimulation of the human keratinocyte cell line - HaCaT with ultraviolet B radiation upregulated the production of IL-18 (198). Several studies have strongly indicated that IL-18 is a strong biomarker for clinical psoriasis symptoms (139, 140, 199). Overall, these data may indicate the potential role of IL-18 in psoriasis therapy.

### Systemic lupus erythematosus (SLE)

SLE is an autoimmune disease characterized by B cell hyperactivity, antibody secretion, and organ damage (200). Studies have continued to show conflicting results on the relationship between IL-18 and SLE, although more recently accumulating evidence reveal that IL-18 may play a vital role in SLE pathogenesis (128). Lupus disease was exacerbated when exogenous IL-18 was administered to MRL/lpr mice but was suppressed following the treatment with anti-IL 18 (201). Particularly, in MRL-Lpr/lpr mice model of SLE, mice administered with cDNA vector expressing IL-18 developed auto-antibodies to IL-18 and had suppressed IFN-γ, milder kidney damage, and less mortality compared to the control mice (202). In agreement, human studies reveal that patients with lupus nephritis had increased concentration of serum IL-18, and kidney biopsies showed IL-18 positive glomeruli compared to normal subjects (8). Several meta-analyses have validated the claim that circulating levels of IL-18 is higher in SLE patients, which suggests the role of IL-18 in the pathogenesis of SLE (202, 203).

### Adult Still’s Onset Disease (AOSD)

AOSD is an auto-inflammatory systemic disease of unknown etiology and characterized by spiking fever, rash, arthritis, leukocytosis, and other signs (204, 205). IL-18 is one of the likely inflammatory agents involved in the pathogenesis of AOSD. Its overexpression has been linked to driving the inflammatory process among other immunological factors. Particularly, high concentrations of IL-18 have been described in AOSD and were correlated with laboratory markers of the disease (129, 206). It was indicated that free IL-18 serum concentrations are significantly higher in AOSD patients compared to either healthy or disease controls including RA, SLE, axial spondyloarthritis and psoriatic arthritis. The free IL-18 levels correlated with AOSD activity suggesting that IL-18 may represent a potential target for the treatment of AOSD. In another study, the sera levels of IL-18 of 26 Italian patients with AOSD was investigated to assess the role of IL-18 cytokine as a disease marker and compared with that of 21 patients with RA, 21 patients with Sjogren’s syndrome, 20 patients with SLE and 21 healthy controls (205). Herein, IL-18 serum levels were significantly higher in patients with active AOSD than non-active as well as control groups. More so, IL-18 serum levels significantly correlated with disease activities and other laboratory parameters such as ferritin and C-reactive protein suggesting the possible targeting of IL-18 cytokine as a therapeutic option because of the role it plays in the disease state (207–210).

### Polymyositis and Dermatomyositis (PM and DM)

PM and DM are inflammatory myopathies characterized by muscle weakness, suppressed muscle endurance and skin involvement (DM only) (145, 211). Generally, the pathologies of DM and PM are different. DM is thought to arise from CD4^+^T- and B- cells mediated inflammation while PM is considered to result from autoreactive cytotoxic T cells which may mediate cytotoxic activities against auto-antigens (212, 213). In one study to investigate IL-18 expression in symptomatic and asymptomatic muscle tissues of patients with PM and DM, 2 cohorts of patients were used (145). One cohort consisted of 10 new-onset myositis patients and IL-18 expression levels were compared between the muscle biopsies results of symptomatic and asymptomatic patients before treatment. In the second cohort, 10 patients with repeated muscle biopsies before and 8 months after treatment with immunosuppressive therapy were recruited. Results indicated that the expression of total IL-18 in muscle tissues from new-onset patients (symptomatic and asymptomatic) were significantly higher compared to healthy controls. IL-18 total expression levels were lower in biopsies from patients receiving immunosuppressive treatment compared to other patients. The results indicate that IL-18 is highly expressed in muscle tissues in inflammatory myopathies. In another study to ascertain the involvement of IL-18 in PM and DM inflammation, 33 patients with DM and 16 patients with PM were enrolled in the study (there were some patients with interstitial lung disease in both groups) (130). It was revealed that serum IL-18 levels were significantly higher in DM and PM patients compared with healthy controls. These authors along with others (145, 213, 214) concluded that serum IL-18 levels were strikingly elevated in DM and PM patients and particularly in DM patients complicated with interstitial lung disease. IL-18 levels also indicate clinical severity of dermatomyositis (215) with recent findings reporting that IL-18 containing 5-gene region can be used to differentiate histologically identical dermatomyositis and other skin lesions (216).

## Monoclonal antibodies and drugs targeting IL-18

Targeting IL-18 appears like an obvious potential therapy due to its role in inflammation. The cytokine IL-18 could be blocked using monoclonal antibodies (mAbs), by inhibiting its production in cells, its secretion from cells or molecular binding blockade using binding proteins or antibodies (5, 217). **Table 3** shows some clinical trials targeting IL-18 in the treatment of inflammatory diseases

**Table 3:** Some clinical trials targeting IL-18 in the treatment of inflammatory diseases.

No	Disease examined	Drug type/name/number(company)	Study details	Result/References
1	Type 2 diabetes mellitus	anti-IL-18 monoclonal antibody, GSK1070806 (Glaxosmithcline)	multicenter, randomized, single-blind (sponsor-unblinded), placebo-controlled, parallel-group, phase IIa trial in which 37 obese patients poorly controlled T2DM on metformin monotherapy were randomized	Inhibition of IL-18 did not lead to any improvements in glucose control (218).
2	Rheumatoid arthritis and Psoriasis	Tadekinig alfa, a human recombinant IL-18BP	Adekinig alfa was tested in patients with RA and psoriasis	Free IL-18 levels were not increased in neither RA nor in psoriatic arthritis patients as compared to healthy individual (219).
3	Rheumatoid arthritis	Pralnacasan, an oral caspase 1 inhibitor (Vertex Pharmaceuticals)	Pralnacasan is clinically tested and observed in patients in a phase II RA clinical trial	It was well tolerated and suppressed inflammation but was suspected to promote liver toxicity (220).

### Binding of IL-18

IL-18 binding protein (IL-18BP) is a natural antagonist of IL-18, belonging to the immunoglobulin like receptor type but is not cleaved on cell surface (221). It is a naturally occurring 18 binding agent was first identified in 1999. In mice, two isoforms of IL-18 BP exist (c and d), while a, b, c and d are the human isoforms (222). The IL-18 BPa is the unique and major splice variant of IL-18BP that controls the biological activity of IL-18 by binding with high affinity. Thus, it acts as a soluble decoy receptor (81). Recently resolved crystal structure of the IL-18:IL-18BP complex reveals a sequestration of the IL-18 by IL-18BP influenced by molecular mimicry and steric competition of binding sites compared to IL-18R. The IL-18:IL-18BP also showed a novel higher order 2:2 binding stoichiometry compared to the standard 1:1 binding of IL-18:IL-18R (223). The mechanisms of Th1 responses to microbes may be blunted by the action of IL-18 BP thus reducing autoimmune responses to an infection. Several studies have shown the potential of targeting IL-18BP against IL-18. The suppression of disease severity has been reported in 33 disease models in which the administration of anti-IL18 antibodies or IL-18 BP led to the inhibition of IL-18 (40). Accordingly, in murine models of inflammation including DSS colitis, recombinant IL-18 BP suppressed disease severity (224). Inflammation in the joints of mice treated with IL-18BP were suppressed and mice also exhibited reduced inflammatory infiltration and cartilage destruction as observed in the histopathology analysis. Furthermore, *in vivo* studies revealed that short-term supplementation with IL-18 BP Fc prophylactically protected NOD mice from the acceleration of autoimmune diabetes (18). At low dosing of IL-18 BP, inflammation was suppressed in a model of RA (39). Recently, in a clinical trial using human recombinant IL-18 BP in RA and Psoriasis patients, therapeutic efficacy was not achieved (219). Aside from the use of IL-18 BP for binding IL-18, it could also be bound by anti-IL 18 antibodies. In agreement, the severity of Collagen induced arthritis was reduced significantly in the course of treatment with polyclonal anti-IL-18 antibodies and was more effective compared to IL-18 BP (224). In addition, DSS colitis was minimally suppressed in experimental mice using antimurine IL-18 antiserum (225). In MRL/MpJ-Tnfrsf6^lpr^ mice exhibiting lupus like autoimmune syndrome, vaccination with IL-18 cDNA in order to inhibit IL-18 showed that IFN-y production was suppressed and mice showed less glomerulonephritis and renal damage (202). In mice, a neutralizing IL-18Ra antibody showed significant protective effect on a graft-versus-host disease model (226), a condition characterized by systemic inflammation and multiple organ damage.

### Inhibition of IL-18 production

Caspase 1 appears to be a suitable target to suppress the production of IL-18 and also IL-1β (217). The active form of caspase-1 is a tetramer that cleaves the proform of IL-1β and IL-18 to their mature forms (44), which then leaves the cytosol. A study revealed that IL-1β or IL-18 deficient mice were not completely protected from septic shock unlike caspase-1 deficient mice (227, 228). Similarly, in Caspase-1 deficient mice, the severity of DSS induced colitis was suppressed correlating with IL-18 reduced expression (229). Pralnacasan is an oral caspase 1 inhibitor clinically tested and observed in patients in a phase II RA clinical trial (220). It was well tolerated and suppressed inflammation but was suspected to promote liver toxicity.

Uncontrolled mature IL-18 secretion and IL-1β is responsible for severe autoimmune disorders as they bind to their receptors, initiate several signaling, and ultimately activate NF-kB (230). The antagonism of P2X-7, a purinergic receptor located on the cells of hematopoietic origin, might be a potential target for treating autoimmune diseases with inflammatory origin. This is because of the importance of P2X-7 receptor in the biology and secretion of IL-1β and IL-18. This has been confirmed in P2X-7 deficient mice (217, 231).

### Monoclonal antibodies

Currently, GSK 1070806, a humanized mAb, is in a single-blind randomized placebo-controlled phase 1 trial against IBD. The study aims to investigate the use of the drug in both healthy and obese male subjects for the treatment of IBD as well as its safety, tolerability, pharmacokinetics, and dynamics. In this multicenter, randomized, single-blind, placebo-controlled parallel group phase IIa trial conducted in obese patients of either sex with poorly controlled Type 2 diabetes patients on metformin monotherapy using GSK1070806, although it was well tolerated, IL-18 inhibition did not improve glucose control (218). Recently, a novel anti-human IL-1R7 monoclonal antibody that blocks and suppresses the inflammatory signaling of IL-18 was developed. It acts by reducing IL-18 induced NFkB and IFN-γ activation and IL-6 production in human cell lines. It is important to note that IL-1R7 is a potential virgin therapeutic strategy for the investigation of its clinical potential in treating IL-18 mediated diseases as this area remains to be explored (232).

Another novel development in the generation of IL-18 monoclonal antibodies lies in the identification of a neoepitope that is generated after IL-18 is cleaved by the caspases. This neoepitope - ^37^YFGKLESK^44^ can be used to distinguish between physiological and pathological IL-18 (233). This led to the generation of 2 high affinity antagonistic IL-18 antibodies recognizing epitope ^63^NRPLFEDMT^68^ of the full-length human IL-18 cytokine or recognizing only the neoepitope - ^37^YFGKLESK^44^ in humans (233) or ^36^NFGRLHCTT^44^ in mice (234). This strategy of controlling pathogenic IL-18 sounds promising for therapeutic purposes.

Despite abundant evidence showing the role of IL-18 as a biomarker in several inflammatory and autoimmune conditions, it is not being utilized as a target for biologics to control these conditions.

## Conclusion

Despite a large number of reports that have indicated the indispensable role of IL-18 in autoimmune diseases, many of them are still elusive. For instance, the definitive steps required in IL-18 signaling and activation triggers in different autoimmune diseases are widely unknown. More clinical trials of IL-18 BP and other antibodies are necessary to properly ascertain the role of the cytokine IL-18 in treating diseases. IL-18 plays a vital pathogenic role in diseases by promoting T cell mediated responses and may be Th1 or Th2 related. Many *in vitro* studies, animal models, and some clinical studies support the vital role of IL-18 in Psoriasis, MG, and other diseases. Therefore, in order to gain more insight into the place of IL-BP and other drugs targeting IL-18 for the treatment and control of autoimmune conditions, additional research is required. Finally, we recommend the development of combination drug therapies that specifically focus on IL-18 inhibition in addition to the inhibition of other specific cytokines such as TNF-α, IL-1β, IL-6, or IFN-γ which have been indicated to be strongly involved in the pathogenesis of any of the inflammatory diseases.

## Author contributions

All authors (SAI, SDA, ZZ, TS, MS, SM, and GA) helped conceptualize the manuscript. SAI and SDA wrote first draft of the manuscript, figures and tables. All authors (SAI, SDA, ZZ, TS, MS, SM, and GA) reviewed the manuscript and contributed to edits. All authors contributed to the article and approved the submitted version.

## Conflict of interest

Authors MS and SM were employed by AryoGen Pharmed Inc.

The remaining authors declare that the research was conducted in the absence of any commercial or financial relationships that could be construed as a potential conflict of interest.

## Publisher’s note

All claims expressed in this article are solely those of the authors and do not necessarily represent those of their affiliated organizations, or those of the publisher, the editors and the reviewers. Any product that may be evaluated in this article, or claim that may be made by its manufacturer, is not guaranteed or endorsed by the publisher.
